# Orthodontic emergencies and mental state of Chinese orthodontic patients during the COVID-19 pandemic

**DOI:** 10.1186/s12903-021-01834-3

**Published:** 2021-09-27

**Authors:** Shuqi Quan, Yutong Guo, Jiawei Zhou, Guanning Zhang, Ke Xing, Hongxiang Mei, Juan Li

**Affiliations:** Department of Orthodontics, West China Hospital of Stomatology, West China School of Stomatology Sichuan University, State Key Laboratory of Oral Diseases, 14#, 3rd Section, Renmin South Road, Chengdu, China

**Keywords:** COVID-19, Questionnaire, Orthodontic emergencies

## Abstract

**Background:**

The ongoing COVID-19 pandemic postponed routine follow-up visits of many orthodontic patients, which compromised their treatment process and mental states. This study was aimed to assess orthodontic emergency occurrence and psychological states of Chinese orthodontic patients during this pandemic.

**Methods:**

Orthodontic patients in China were invited to answer an anonymous online questionnaire from February 20, 2020 to March 5, 2020, when routine dental care was suspended in China. The questionnaire included self-assessment of oral hygiene and compliance, orthodontic emergencies, perceptions and feelings about COVID-19 and anxiety self-rating scale, etc. Collected data was statistically analyzed with Chi-square, independent t test and univariable generalized estimating equations regression analysis.

**Results:**

A total of 1078 respondents (292 male; 786 female) from 30 provinces of China were included in this study. About one-third (33.67%) of patients reported that they encountered orthodontic problems during the pandemic. Patients with clear aligners reported fewer orthodontic problems than those with fixed appliances or removable appliances. Female patients, elder patients and patients who encountered orthodontic emergencies were more anxious than other patients.

**Conclusions:**

The compliance and occurrence of orthodontic emergencies differed in patients with different orthodontic appliances. Patients with orthodontic emergencies exhibited higher anxiety states.

## Background

Since December 2019, the coronavirus disease 2019 (COVID-19), caused by severe acute respiratory syndrome coronavirus 2 (SARS-CoV-2) [1], has been rampant in China and even all over the world [2]. On March 11, 2020, the World Health Organization (WHO) declared the COVID-19 a global pandemic [3].

To curb the human-to-human transmission of the COVID-19 pandemic, people were encouraged to stay at home and limit social contacts as far as possible [4] and a series of quarantine policies had been enacted by the Chinese Government since the outbreak.

It is now believed that respiratory droplets and contact transmission are the main interpersonal transmission routes of the COVID-19 [5]. Hence, dental practitioners and patients of dental clinics and hospitals faced a higher risk of cross-infection [6], because of large amount of droplets and aerosols generated during the unique dental procedures. Therefore, dental hospitals and clinics in China suspended general non-emergency dental treatment [7] since late January 2020, and only essential emergency dental services could be provided.

Orthodontic treatment is usually a long-term treatment and requires regular follow-up visits of the patients. However, due to the suspended dental service and the quarantine policies, most of the orthodontic patients had to postpone their routine visits. During this prolonged no follow-up period, they might encounter various orthodontic emergencies, such as brackets and attachments falling off, but could not get in-time assistance. Besides, some orthodontic patients might be concerned about the impact of the pandemic on the course and effectiveness of orthodontic treatment, and suffered from the resulting anxiety. Hence, we conducted this study to investigate orthodontic emergencies of the patients with different orthodontic appliances during the COVID-19 pandemic and their solutions, and to evaluate the psychological state of the patients.

## Methods

### Participant recruitment

All experimental protocol was established according to the ethical guidelines of the Helsinki Declaration and approved by the ethic committee of West China Hospital of Stomatology (Approval no. WCHSIRB-D-2020-068). Informed consent was obtained from individuals before they participated in the survey. For subjects below16, informed consent was accent obtained from their guardians. The cross-sectional survey was adopted in this research. An online questionnaire was distributed to the orthodontic patients via WeChat (Tencent, Shenzhen, China), the most widely used instant messaging communication app in China. The snowball sampling routes were utilized to recruit participants. About 50 orthodontists in public hospitals or dental clinic were invited to help distribute the questionnaires to their patients and help ask their colleagues to send to their patients. The inclusion criteria were as follows: patients undergoing orthodontic treatment; patients aged 8 and above; patients with normal learning abilities that could understand and complete the questionnaire; patients who had informed consent of this survey. The questionnaires were distributed from February 20, 2020 to March 5, 2020, when the routine outpatient service of dental hospitals and clinics has been closed nationwide for nearly a month. The professional online survey platform (www.wjx.cn) was used to collect the questionnaires.

Questionnaires with an answering time less than 104 s (calculated based on the answering time of each sub-item not less than 2 s [8]), or with more than 10 consecutive answers through the reverse designed questions [8, 9] were considered invalid and screened out.

### Measurements

The questionnaire consisted of mainly 5 parts. Part 1 gathered the socio-demographic information and orthodontic status of the respondents, including gender, age, education level, occupation, orthodontic mode, choice of orthodontic office and geographical relationship with the hospital or clinic. Part 2 evaluated the self-management ability of oral health and the compliance and cooperation of the participants compiled by a 5-point Likert scale.

Part 3 investigated the orthodontic problems and emergencies encountered by patients during the pandemic period, such as falling off of brackets and attachments, sliding of arch wires, ligation wire pricking oral mucosa, gum swelling and pain, microimplant loosing and others. The solution methods they adopted and whether their problems had been solved were also assessed in this part.

Part 4 focused on the risk perceptions about the COVID-19 pandemic of the participants, including the evaluation of patients’ knowledge of the pandemic, their concern about the risk of infection in receiving orthodontic treatment during the pandemic, the self-assessment of the impact of the pandemic on the duration and efficacy of their orthodontic treatment as well as the willingness to accept orthodontic treatment during the COVID-19 pandemic.

Part 5 was made up with the Chinese vision of Self-Rating Anxiety Scale (SAS) compiled by William W.K., Zung et al. [10], aimed to measure the psychological state, especially the anxiety state of orthodontic patients. This standardized questionnaire consisted of 20 declarative sentences and each item required 4 points for evaluation, ranging from 1 point meaning never or seldom to 4 point meaning all of the time. Domestic studies have found that the Chinese version of the questionnaire has good reliability and validity [11], which means it is suitable for Chinese subjects.

### Statistics analysis

Descriptive statistics were used to present sociodemographic characteristics, orthodontic status and emergencies of patients. Continuous variables were calculated by mean and standard deviation, while categorical variables were calculated by frequency and percentage.

Cronbach's Alpha and KMO and Bartlett’s test were used respectively to verify the reliability and validity of data from self-made scale. The comparison of means of different groups was performed by the t-test, and chi-square test was used to compare rates between different sample. Univariable generalized estimating equations (GEE) regression analyses were used to analyze the related factors of occurrence of orthodontic emergencies and anxious psychological state. Data analysis was performed with SPSS Statistics version 26 (IBM Corp, Armonk, NY). Two-sided P<0.05 indicates the statistically significant.

## Results

A total of 1239 questionnaires were collected. According to predetermined eligibility criteria, 161 questionnaires were deemed invalid and 1078 questionnaires from 1078 respondents were finally included.

### Socio-demographic variables and orthodontic information

Table 1 illustrates the demographics and orthodontic information of the patients answering the questionnaires. The majority of the respondents were female. Age of the patients ranged from 8 to 65, with an average age of 22.59 (SD 8.277). The respondents represented 30 province-level regions in China. Students made up 51.58% of the respondents.

**Table 1:** Demographics and orthodontic information of the patients answered the questionnaires

Characteristics	N	Percent (%)
Demographics		
Age group		
8–12	43	4.0
12–18	305	28.3
18–30	531	49.3
30–45	184	17.1
45–65	15	1.4
Gender		
Male	292	27.1
Female	786	72.9
Profession		
Student	556	51.6
Teacher	53	4.9
Medical personnel	67	6.2
Employee of enterprises and institutions	158	14.7
Government staff	39	3.6
Freelancer	69	6.4
Others	136	12.6
Education background		
Postgraduate	129	12.0
Tertiary school	548	50.8
Secondary school	321	29.8
Primary school	80	7.4
Orthodontic		
Orthodontic appliances		
Fixed appliances	581	53.9
Clear aligners	347	32.2
Removable appliances	150	13.9
Geographical relationship with the hospital or clinic		
In the same city	744	69.0
In the same province (except the same city)	223	20.7
In different provinces	111	10.3
Dental office		
Orthodontic department in stomatological hospital	623	57.8
Stomatology department in general hospital	133	12.3
Chain dental clinic	103	9.6
Individual dental clinic	219	20.3
Total	1078	100.0

In regard to orthodontic status, more than half of the respondents were wearing fixed orthodontic appliances, while 32.19% were being treated with clear aligners, and 13.91% were treated with removable appliances. In the option of hospital or clinic, 70.1% respondents registered with public hospital to receive orthodontic treatment.

### Evaluation of oral hygiene, compliance and cooperation

The Cronbach's Alpha of oral hygiene scale is 0.795, which shows the data is of high reliability [12]. KMO and Bartlett’s test was used to verify the validity, and the KMO value was 0.773(between 0.7 and 0.8), which shows a good validity of the research data.

Most of the patients considered their oral hygiene to be good (83.67%) and gum bleeding was less likely to happen (24.58%). Specific to each oral hygiene measure, 30.71% patients reported they brushed their teeth carefully after each meal. However, fewer patients insisted on using dental floss or oral irrigator. Only 23.28% of them were fully consistent with insisting on using oral irrigator, and 16.33% had never used dental floss or oral irrigator.

For the compliance and cooperation of the orthodontic patients, 76.67% of the patients with fixed appliance considered themselves to be very cooperative with orthodontic treatment, and 66.96% with clear aligners, and 68.53% with removable appliances.

### Problems encountered during orthodontic treatment

Among the 1078 patients participating in the survey, 33.67% were reported to have encountered problems related to orthodontics during the pandemic.

Statistical analysis shows the score of oral hygiene and the different orthodontic modes are associated with the emergency of orthodontic problems (Table 2). Patients with clear aligners had fewer orthodontic emergencies, with the percentage of 23.9, compared with 38.9% of the patients with fixed appliances and 36.0% patients with removable appliances.

**Table 2:** Univariable generalized estimating equations (GEE) regression analyses for the orthodontic emergencies

Variables	Or	95% CL	P-value
Orthodontic appliances			
Fixed orthodontic appliances	Reference		
Clear aligners	0.499	(0.367, 0.679)	< 0.0001
Removable appliances	0.858	(0.581, 1.268)	0.443
Oral hygiene			0.009

**Fig. 1:**
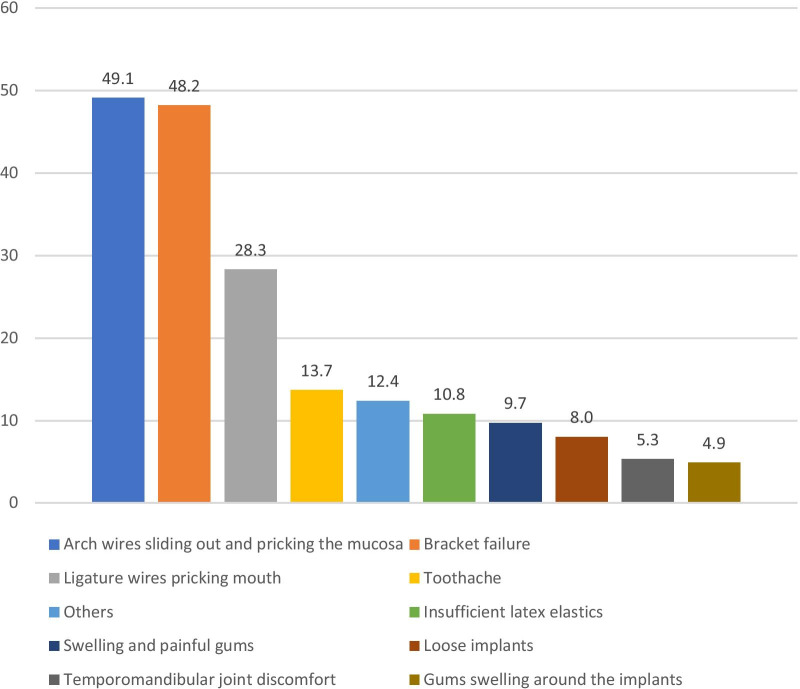
Most frequent problems encountered by patients with fixed appliances (%)

Among patients had orthodontic problems with fixed appliances, the incidence of mucosal problems caused by bracket shedding and arch wires slipping out were the highest, both accounting for about 50% (Fig. 1). Mucosal problems caused by ligation wire pricking the mouth accounted for about 28.32%.

**Fig. 2:**
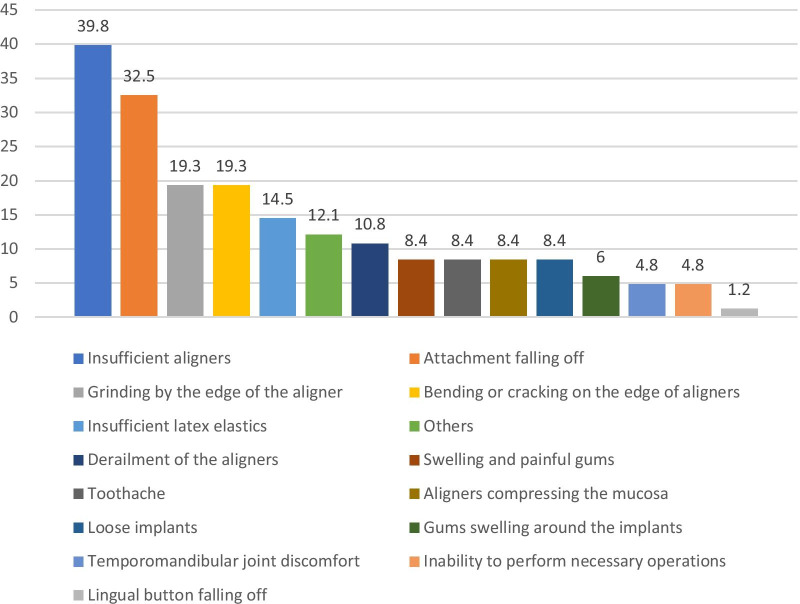
Most frequent problems encountered by patients with clear aligners (%)

Most frequent orthodontic problems encountered by patients with clear aligners are not carrying enough aligners (39.76%), followed by falling off of attachments (32.53%), grinding by the edge of the aligners (19.28%), bending or cracking on the edge of the aligners (19.28%), insufficient latex elastic (14.46%) and other problems (Fig. 2).

**Fig. 3:**
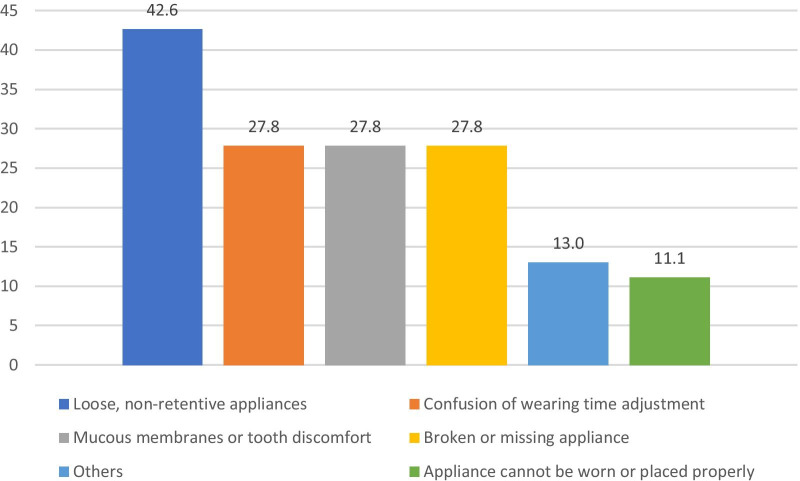
Most frequent problems encountered by patients with removable appliances (%)

Most common problem encountered by patients wearing removable appliances during the pandemic is that the appliances were easy to become loose and dislocate after being worn (42.59%). The percentage of the patients did not know whether it is necessary to adjust the wearing time, the patients with mucosal or dental discomfort caused by the appliances and the patients who reported their appliances were broken or missing were all 27.78. Only 11.11% of the patients reported that their appliances cannot be worn or placed at all (Fig. 3).

### Solution mode

Faced with orthodontic emergencies, more than half (55.10%) of the patients contacted their attending doctors. 33.33% of the patients thought the problem was tolerable and did not take any measures, and 23.14% of the patients indicated that they could solve it by themselves according to their experiences or common sense. The other ways in which patients solved their orthodontic problems were going to the hospital for emergency treatment (13.77%), searching for related online science articles (7.71%), consulting through the online health platforms (5.78%), and others (5.23%). Still 7.16% of patients suffered from the orthodontic emergencies but no attempt was made to solve it.

Through the measures above, only 24.52% of patients’ problems were solved, and 30.58% of patients reported that temporary measures were taken to improve the problems, but they still needed a follow-up visit to completely solve the problems. Besides, 21.21% of patients just understood the problem-related knowledge but did not help, and 23.69% of patients’ problems were not resolved at all.

### Risk perception about the COVID-19 pandemic

For the knowledge of the pandemic, there were no significant differences among subjects in different genders, occupations, and educational backgrounds. However, participants in 30–45 age group had a significantly higher understanding of the pandemic than which in 12–18 and 18–30 age groups.

About the reasons for difficulties of orthodontic follow-up visits due to the pandemic, 67.90% of patients were worried about the risk of infection, 59.28% were because the hospital or clinic did not open, 25.05% chose the reason that it was hard to go to the hospital due to traffic factors, and only 11.50% of them thought it had little effect.

Regarding the closure of dental apartments during the pandemic, 73.93% of the patients expressed complete understanding and support, 25.32% expressed that they could understand, only 8 patients (0.74%) expressed some incomprehension, and 0 persons expressed completely couldn’t understand.

About the degree of concern about the infection risk in orthodontic treatment (1–5), more than half (54.91%) of patients were quite worried about it, while 9.28% were not worried at all.

Regarding the patients’ willingness to accept orthodontic treatment after the resumption of dental service, patients with orthodontic emergencies were more likely to undergo orthodontic follow-up visits as soon as possible than those without orthodontic emergencies (P < 0.0001). Compared with patients with clear aligners and patients with removable appliances, patients with fixed appliances prefer to take follow-up visits as soon as possible (P = 0.008).

As is shown in Table 3, for patients’ self-assessment about the influence of the pandemic on their orthodontic treatment, most of the patients thought it had a little or moderate impact on the duration and efficacy.

**Table 3:** Patients’ self-assessment of the impact of pandemic on orthodontic treatment

Variables	Category	N	Percent (%)
Duration	1	156	14.5
	2	403	37.4
	3	275	25.5
	4	158	14.7
	5	86	8.0
Efficacy	1	245	22.7
	2	379	35.2
	3	253	23.5
	4	139	12.9
	5	62	5.8

The Category 1 = Impact not at all, 2 = Impact a little, 3 = Impact moderately, 4 = Impact much, 5 = Impact very much

### Mental state

The SAS anxiety scale was used to evaluate the mental state of patients during the period of pandemic when they could not undergo regular orthodontic follow-up visits. The average SAS score of all subjects was 36.26 (SD 7.25).

The average SAS score of patients without orthodontic emergencies was 35.08 (SD 6.41), where 11.3% of the patients were anxious, while the average score of patients with orthodontic emergencies was 38.60 (SD 8.17), and only 2.4% were anxious.

Table 4 shows the GEE regression analyses results for the anxiety scores of orthodontic patients. For multivariable analysis, female respondents (P = 0.046), patients aged above 45 (P = 0.040) were more anxious, while patients with invisible aligner (P = 0.020) were less. Patients who encountered orthodontic emergencies during the pandemic exhibited a significantly higher anxiety level than those who did not have orthodontic emergencies (P < 0.0001).

**Table 4:** Multivariable generalized estimating equations (GEE) regression analyses for anxiety scores of orthodontic patients

Variable	Or	95% CL	P-value
Demographics			
Gender			
Male	Reference		
Female	2.686	(1.014, 7.104)	0.046
Age group			
8–12	Reference		
12–18	2.620	(0.298, 23.043)	0.385
18–30	18.530	(0.826, 415.473)	0.066
30–45	7.725	(0.250, 239.159)	0.243
45–65	260.450	(1.293, 52,452.538)	0.040
Orthodontic			
Orthodontic appliances			
Fixed orthodontic appliances	Reference		
Clear aligners	0.319	(0.122, 0.836)	0.020
Removable appliances	1.433	(0.353,5.812)	0.615
Orthodontic emergencies			
Encountered	Reference		
Not encountered	0.063	(0.024, 0.166)	< 0.0001

## Discussion

Due to the Covid-19 pandemic, dental hospitals and clinics in most parts of China had been closed since late January 2020, and only reserved essential dental emergency departments. However, orthodontic treatment is a long-term treatment requiring regular follow-up visits, which were almost all delayed in those days. Our study indicates that the pandemic does have some impacts on orthodontic patients.

Our study suggests that patients with fixed appliances had a higher degree of self-evaluation of orthodontic cooperation, which might be related to the fixity of the appliances and less operation requirement for patients. Generally, patients with fixed appliances are only required to wear latex elastics or even not, while invisible aligners and removable appliances require more complicated and frequent cooperation, and usually they can be picked and worn by patients themselves easily. Only two-thirds of the patients with invisible aligners or removable appliances reported they could well cooperate. This is similar to the results of previous systematic reviews [13]. Patients had poor compliance with removable orthodontic appliances and adjuncts, and they often overestimated their wearing time. The actual objective cooperation of orthodontic patients may be even less ideal.

In this study, the most common problems reported by patients with fixed orthodontic appliances are arch wires pricking mucosa and bracket failure. This is in line with previous studies [14, 15]. The systematic review of Almosa et al. [16] reported the incidence of brackets detachment were 0.6% to 9.6% in most study. A higher incidence of brackets detachment was presented during this long no follow-up visits period caused by the pandemic. According to the advice of Caprioglio et al. [17], temporary measures can be taken by patients or their family members under the guidance of orthodontists to reduce the damage to mucosal tissue, such as using nail clippers to cut off the arch wires, or applying mucosal protective wax to protect mucous membrane from being damaged by sharp parts of appliances.

Compared with fixed and removable appliances, clear aligners caused fewer emergencies during this period, which indicates that clear aligners have advantages for patients who live far from hospitals or have to schedule the follow-up visit in a long period for some reason. The most common problem with clear aligners is not carrying enough aligners because of the outbreak of the pandemic.

The main problems with removable appliances were the loosening or dislocation of appliances after they were put on. This may be related to the fact that removable appliances are often used in growing young patients. Growth of patients’ jaws and alveolar bones, or incorrect use of the appliances during the pandemic without being corrected by orthodontists in time, resulting in difficulties in positioning the appliances. Proper design and regular follow-up visits for the adjustment of retention components [18] are the useful methods to enhance the stability of removable appliances.

About half of patients encountered orthodontic problems in this study reported they contacted their attending doctors. This proportion is less than the findings of Bustati et al. [19], which reported 74% orthodontic patients communicated with their orthodontists at least once during the COVID-19 pandemic in Syria. Allowing patients to have efficient communication with orthodontists during this no follow-up period is an effective way to solve orthodontic problems. Orthodontic teleassistance can be used as a means to help orthodontists deal with patients’ emergencies and also a monitoring method during the non-revisiting period [20]. Patients can take regular photos or make video calls with orthodontists for guidance. To protect the privacy of orthodontists, the work number can be utilized to keep in touch with patients at appropriate time, such as working hours [21]. Bustati et al. [19] and Cotrin et al. [14] reported most of the orthodontic patients utilized the mobile phone applications to get in touch with their orthodontists during the pandemic. Besides, the use of social media applications has been documented to be useful in improving oral hygiene compliance [22] and post-orthodontic compliance [23] of orthodontic patients. This indicates the important role of mobile phone applications in the communication between patients and orthodontics.

Our data suggest that female patients suffered a greater anxiety state during this no follow-up period, which corresponds to previous studies [24]. This can partly be explained by that female gender suffered higher levels of anxiety and a greater psychological impact of the pandemic among general population in China [25]. Also, Peloso et al. [26] revealed that female patients were more anxious and afraid to go to a dental appointment. Besides, patients aged 45–65 were found to be more anxious during the pandemic.

It is notable that the anxiety state of patients encountered orthodontic emergencies was significantly higher than patients without orthodontic emergencies. Orthodontists need to pay attention to the psychological states of patients either during the quarantine or after the routine follow-up visits resumed, and offer corresponding advice and comfort to patients, especially those who encountered with orthodontic emergencies. Moreover, appropriate measures could be adopted by the orthodontics to reduce the incidence of orthodontic emergencies, in order to relieve the anxiety of patients.

The main limitation of the study is that since this questionnaire was sent online, most of the respondents were young people who used smartphones. More than half of the respondents aged 18–30, which may affect the generalizability of the results.

## Conclusion

The results of this study show that oral hygiene and different orthodontic modes are associated with the incidence of orthodontic problems patients encountered during the cessation of regular orthodontic follow-up visits for the COVID-19 pandemic in China. Patients with clear aligners have fewer orthodontic emergencies than those with fixed appliances and removable appliances. Female patients, patients aged above 45 and those who encountered orthodontic emergencies exhibited more anxious mental states.

## Data Availability

The datasets generated during and analyzed during the current study are not publicly available considering that individual privacy might be compromised, but are available from the corresponding author on reasonable request.

## Abbreviations

COVID-19: Coronavirus disease 2019
SAS: Self-Rating Anxiety Scale
KMO: Kaiser–Meyer–Olkin
GEE: Generalized estimating equations
SD: Standard deviation
